# Association between *INHA* gene polymorphisms and litter size in Hainan black goats

**DOI:** 10.7717/peerj.15381

**Published:** 2023-05-09

**Authors:** Zhengyu Bian, Kunpeng Li, Si Chen, Churiga Man, Fengyang Wang, Lianbin Li

**Affiliations:** Key Laboratory of Tropical Animal Breeding and Epidemic Disease Research of Hainan Province, School of Animal Science and Technology, Hainan University, Haikou, China

**Keywords:** Hainan black goats, *INHA* gene, Polymorphism, Reproductive performance, Litter size

## Abstract

**Background:**

The inhibin alpha (*INHA*) gene is one of the important genes affecting the reproductive traits of animals. Hainan black goats are the main goat breed in Hainan Island (China), whose development is limited by low reproductive performance. However, the relationship between *INHA* gene and the reproductive performance of Hainan black goats is still unclear. Therefore, the purpose of this work was to explore the effect of *INHA* gene polymorphisms on the litter size of Hainan black goats.

**Methods:**

Single nucleotide polymorphisms (SNPs) of *INHA* were detected, and the genetic parameters and haplotype frequency of these SNPs were calculated and association analysis was performed for these SNPs with the litter size. Finally, the SNP with significant correlations to litter size was analyzed by Bioinformatics tools.

**Results:**

The results showed that the litter size of individuals with the *AC* genotype at loci g.28317663A>C of *INHA* gene was significantly higher than those with the *AA* genotype. This SNP changed the amino acid sequence, which may affect the function of *INHA* protein by affecting its structure. Our results suggest that g.28317663A>C loci may serve as a potential molecular marker for improving the reproductive traits in Hainan black goats.

## Introduction

As one of the earliest domesticated livestock species, goats are closely related to human activities (Tabbaa & Al-Atiyat, 2009). Hainan black goats are a unique local breed in Hainan and the only breed on the tropical island in China (Hu et al., 2016). Hainan black goats have a good tolerance to hot and humid environment, and their meat is delicious and popular among people in southern China (Shi et al., 2020). Hainan black goats usually attain sexual maturity at 4–6 months of age, and first breed at 7–8 months of age. They normally produce a single kid per year and sometimes three kids in 2 years (Hua et al., 2019). Due to their poor reproductive performance, their development is currently limited.

Kidding traits are affected by several factors (genetics, environment, nutritional status, *etc*.). Genetic factors are the most critical factors (González-Rodríguez et al., 2018; Ofori & Hagan, 2020; Zheng et al., 2020). The *INHA* gene has been shown to be associated with the kidding ability of many animals, such as the Suhuai pigs and Dazu black goats (Liu et al., 2017a; Wang et al., 2021). *INHA* gene is the main gene controlling the biological activity of inhibin. Inhibin is mainly secreted by male testicular cells and female ovarian granulosa cells (Cai et al., 2011; Cui et al., 2020). Inhibin regulates reproductive functions through endocrine, paracrine, and autocrine modes, specifically acting on the pituitary gland. It inhibits the secretion of follicle-stimulating hormone (*FSH*), and locally regulates the production of estrogen (*E*) (Burger, 1988). High levels of *FSH* and *E* will lead to greater ovulation in animals and thus more chances of multiple births, which may be an important reason why *INHA* affects litter size. However, *INHA* gene polymorphism in Hainan black goats has not been studied.

When considering candidate genes involved in reproductive performance, it is important to detect single nucleotide polymorphisms (SNPs) and to analyze the associations between these SNPs and reproductive traits. Therefore, this study aims to detect *INHA* gene polymorphisms in Hainan black goats by sequencing and analyzing the relationship between *INHA* gene variants and litter size.

## Materials and Methods

### Animals, sample collection, and DNA preparation

All of the conducted procedures were approved by the Hainan University Institutional Animal Use and Care Committee (No. HNUAUCC-2022-000121). Jugular blood samples of 211 Hainan black does were collected from Hainan Chuxin Animal Husbandry Co,. Ltd. (Ding’an, Hainan, China) in two different locations. There were 90 Hainan black does with records of kidding, of which 37 had two kids per litter and 53 had single kid per litter. The remaining 121 Hainan black does were randomly selected in the farms. The genomic DNA of these ewes was extracted with a blood genomic DNA extraction kit (Tiangen Biotech Co., Ltd., Beijing, China).

### Amplification of the *INHA* coding sequence

The full-length exon sequence (accession number: NC_030809.1) of the *INHA* gene of Hainan black goats was used for designing four pairs of primers using the Primer 3Plus web-site according to the *INHA* gene sequence (GENE ID: 100861261) in NCBI (Table 1). We divided 211 DNA samples of Hainan black does into three groups consisting of 70/70/71 samples. DNA samples in each group were pooled and PCR conducted using primers 1, 2, 3, 4. Primer 1 is used to amplify exon 1, and primer 2–4 is used to amplify exon 2.

**Table 1:** Sequences and characteristics of primers used for amplifying Hainan black goats *INHA* gene.

Primer name	Primer sequence (5′–3′)	Position in goat sequence	Annealing temperture/°C	Amplified fragment/bp
*INHA*-1-F	AGAGATAGGAGGTCTCAATG	*INHA*-exon 1 (−198…406)	56	605
*INHA*-1-R	AGGAAGGTTTAGTAACTGGT			
*INHA*-2-F	AGAGGGGTCCCAGGTTTT	*INHA*-exon 2 (-59…540)	56	600
*INHA*-2-R	GCTCCTGGAAAGAGATATTGA			
*INHA*-3-F	GTTGTCCTCTCTGTTCCTG	*INHA*-exon 2 (316…821)	56	506
*INHA*-3-R	TTAGATGCAAGCACAGTGC			
*INHA*-4-F	ATCTTCCACTACTGTCAC	INHA-exon 2 (579…1010)	56	432
*INHA*-4-R	CACTTATCAGAGAAGCTTG			
*INHA*-5-F	GGACAGACAGGAGACCACT	*INHA*-exon 2 (131…910)	59	780
*INHA*-5-R	GCCTCTGAGCAGAGAGGAG			

**Note:**

The numbers in parentheses start with the first base of the corresponding CDS region of *INHA* gene.

### Genotyping of *INHA* polymorphism

The gene sequence was detected by Shanghai Shengong Bioengineering Co., LTD., and then visualized using SnapGene software. The genotype was determined by the visual sequencing peak. In pooled sequencing, we found the SNPs in both exon 1 and 2. For the one SNP on exon 1, we used primer *INHA*-1 for amplification. The multiple SNPs on exon 2 spanned the fragment of primers *INHA*-2, 3, and 4, so we redesigned primer *INHA*-5 to amplify these SNPs (Table 1). Finally, the distribution of the SNP loci was compared and analyzed. The sequencing data of *INHA* gene has been uploaded in the GenBank database with the accession number (OQ461736–OQ462157).

### Statistical analysis

Popgen32 software was used to count the genotype frequency, allele frequency, observed heterozygosity (Ho), expected heterozygosity (He), effective allele number (Ne), and polymorphism information content (PIC), and to conduct a Hardy Weinberg equilibrium test. Haploview4.2 software was used to analyze the haplotypes and linkage disequilibrium (LD) of the SNPs. At the same time, the same general linear model (GLM) program of SPSS 19.0 software was used to analyze the influence of genotypes and haplotypes on the litter size of Hainan black goats. The models was as follows:

$\rm{Yij=\mu + Gi + eij}$

where Yij is the litter size phenotype of the individual Hainan black goats, μ is the population mean, Gi is the effect of genotype or haplotype, and eij is the random error effect.

### Bioinformatics analysis

First, the integral and coding sequences of gene were obtained from NCBI (https://www.ncbi.nlm.nih.gov/). Then the secondary and the tertiary structures of the *INHA* protein and its variant, which was significantly associated with litter size, were predicted by the online websites PRABI (http://www.prabi.fr/) (Combet et al., 2000) and SWISS (https://swissmodel.expasy.org/), respectively (Waterhouse et al., 2018).

## Results

### *INHA* gene specific fragment

**Figure 1:**
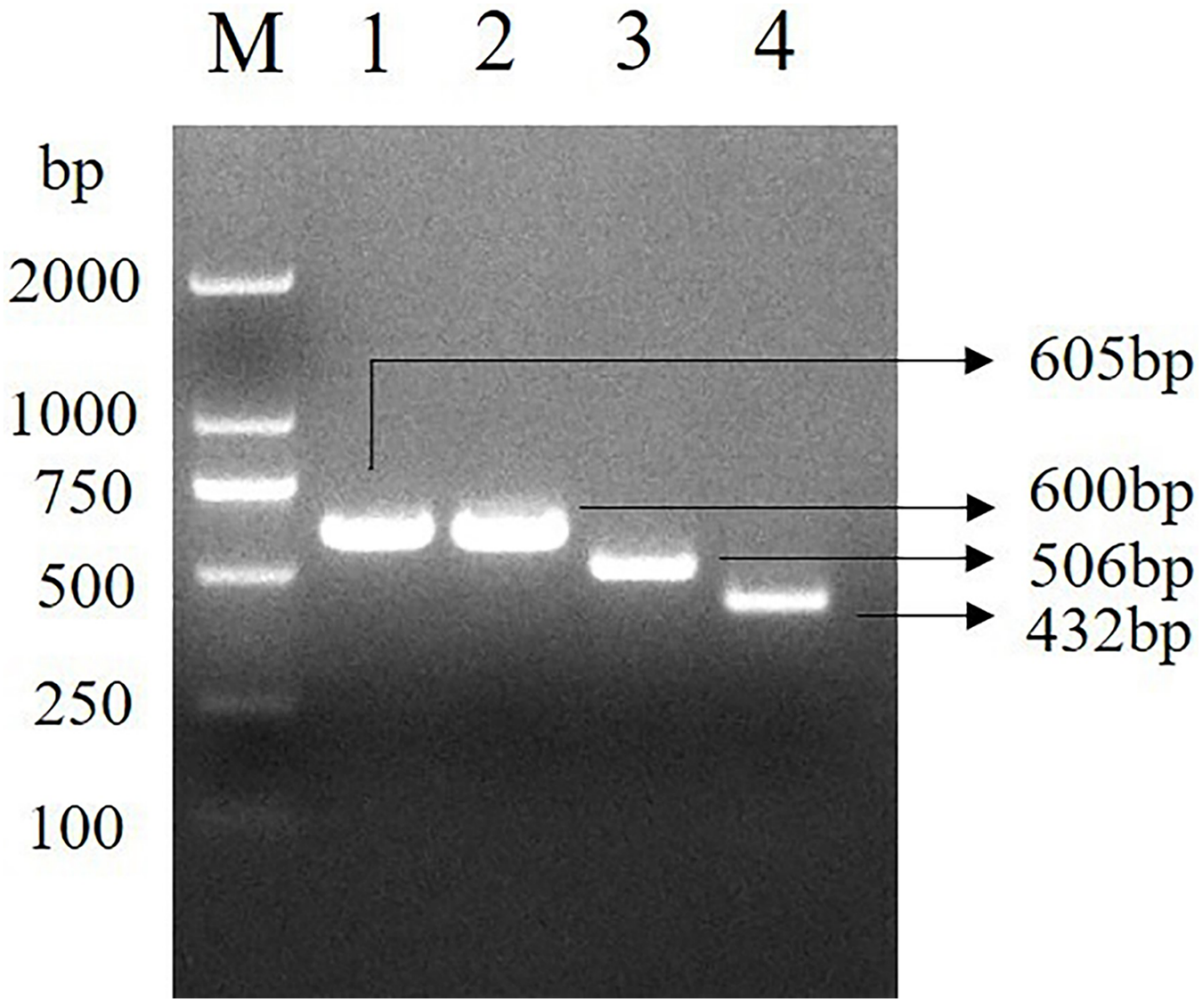
*INHA* gene specific fragment. The electrophoresis results of PCR amplified fragments of the *INHA* gene. M. D2000 DNA Marker; 1. *INHA*-1-F/*INHA*-1-R primer amplified fragment; 2. *INHA*-2-F/*INHA*-2-R primer amplified fragment; 3. *INHA*-3-F/*INHA*-3-R primer amplified fragment; 4. *INHA*-4-F/*INHA*-4-R primer amplified fragment.

Four pairs of primers were used to amplify the *INHA* gene of Hainan black goats, and the four DNA sequence fragments of 605, 600, 506 and 432 bp were obtained (Fig. 1). The amplified fragment was consistent with the target fragment and could be analyzed directly by sequencing.

### SNPs identified by sequencing

**Figure 2:**
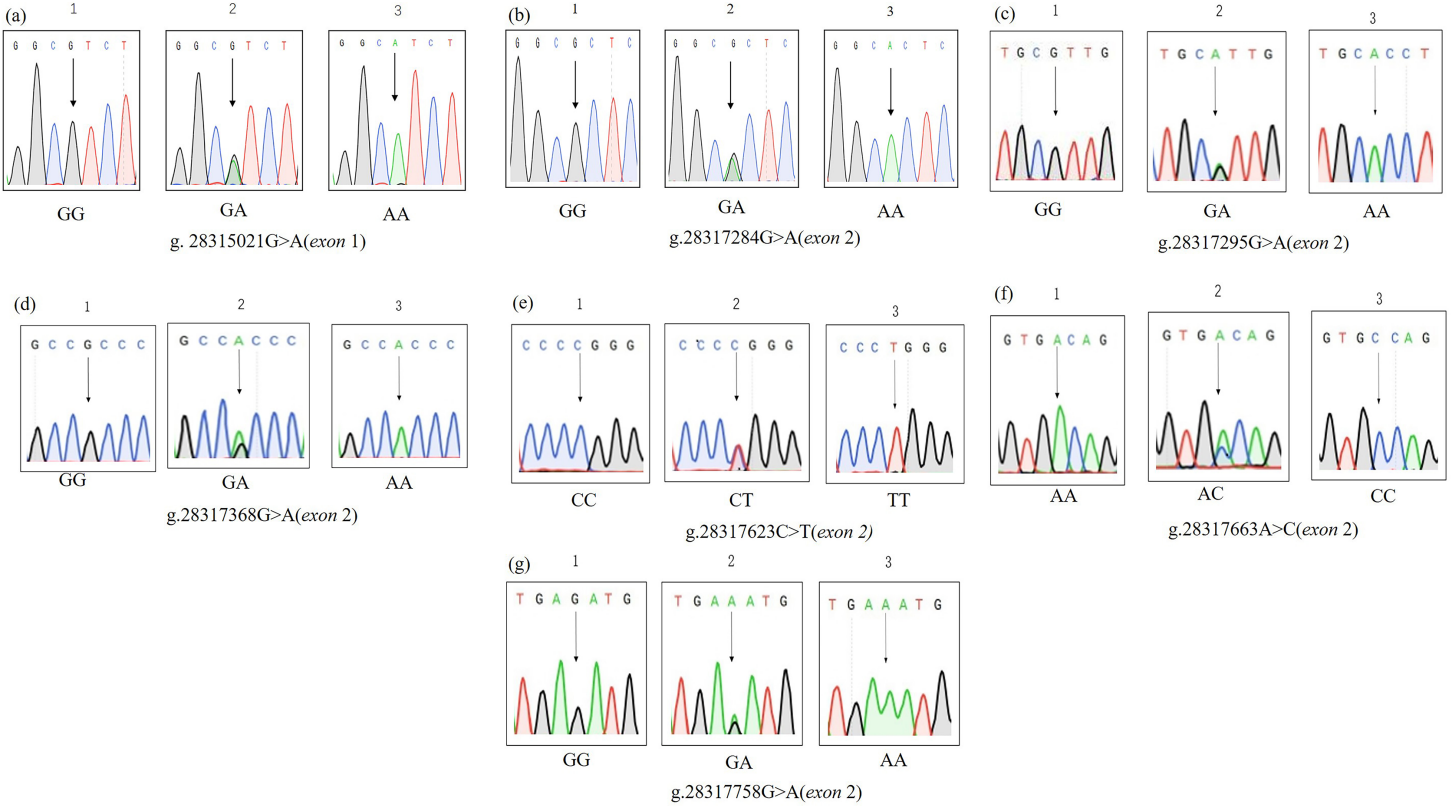
(A–G) SNPs identified by sequencing. SNPs distribution of the *INHA* gene in Hainan black goats.

According to the pooled sequencing results, we found seven SNPs. One SNP was located in exon 1, and the other six SNPs were located in exon 2. Since the six SNPs in exon 2 span fragments of primers *INHA*-2, 3, and 4, we redesigned primer *INHA*-5 (Material S1) to amplify these SNPs. The PCR products were sequenced, and the sequences of different genotypes are shown (Fig. 2). Among them, g.28315021G>A, g.28317295G>A and g.28317663A>C resulted in amino acid sequence changes (Table 2).

**Table 2:** Information content of seven polymorphism loci in the *INHA* gene of Hainan black goats.

Mutation type	Mutation location	Mutation region	Amino acid change
G/A	g.28315021	Exon 1	(Arg→His)
G/A	g.28317284	Exon 2	(Ala→Ala)
G/A	g.28317295	Exon 2	(Arg→His)
G/A	g.28317368	Exon 2	(Pro→Pro)
C/T	g.28317623	Exon 2	(Pro→Pro)
A/C	g.28317663	Exon 2	(Thr→Pro)
G/A	g.28317758	Exon 2	(Glu→Glu)

### Polymorphism analysis of *INHA* gene in Hainan black goats

Genetic analysis of Hainan black goats showed that all SNPs loci were in Hardy-Weinberg equilibrium (*p* > 0.05). The g.28315021G>A, g.28317284G>A, g.28317295G>A, g.28317623C>T, and g.28317663A>C sites were in a low polymorphic information content state (PIC < 0.25). The g.28317368G>A and g.28317758G>A sites were in a moderate polymorphic information content state (0.25 < PIC < 0.50). The results show that these two loci have strong selection potential in Hainan black goats, and their genetic diversity is rich. The homozygosity of all loci was higher than heterozygosity (Table 3).

**Table 3:** Information content of seven polymorphic loci in the *INHA* gene of Hainan black goats.

Locus	Genotype			Allele frequency		He	Ne	PIC	$\chi^{2}$ test
g.28315021G>A	*GG*	*GA*	*AA*	G	A	0.244	1.323	0.241	0.013
	155	52	4	0.86	0.14				
g.28317284G>A	*GG*	*GA*	*AA*	G	A	0.156	1.185	0.166	0.196
	176	34	1	0.91	0.09				
g.28317295G>A	*GG*	*GA*	*AA*	G	A	0.202	1.253	0.196	0.048
	166	42	3	0.89	0.11				
g.28317368G>A	*GG*	*GA*	*AA*	G	A	0.470	1.886	0.471	2.293
	35	89	87	0.38	0.62				
g.28317623C>T	*CC*	*CT*	*TT*	C	T	0.095	1.105	0.095	0.555
	191	19	1	0.95	0.05				
g.28317663A>C	*AA*	*AC*	*CC*	A	C	0.234	1.305	0.241	1.126
	156	53	2	0.86	0.14				
g.28317758G>A	*GG*	*GA*	*AA*	G	A	0.283	1.395	0.282	3.68
	149	52	10	0.83	0.17				

**Note:**

He is the expected heterozygosity; Ne is the number of effective alleles. $\chi^{2}$0.05 (df = 1) = 3.84.

### Association of *INHA* gene polymorphism and litter size in Hainan black goats

The results showed that the genotypes of 90 Hainan black goats at locus g.28317663 were significantly correlated with litter size. In the Hainan black goat breed, the does with *AC* genotype had greater litter size than those with *AA* genotypes (*p* = 0.046) (Table 4).

**Table 4:** Association of *INHA* gene polymorphism and litter size in Hainan black goats.

Locus	Genotype	Number of samples	Litter size (Mean ± SD)
g.28315021G>A	GG	73	1.397 ± 0.492
	GA	17	1.470 ± 0.515
g.28317284G>A	GG	75	1.427 ± 0.498
	GA	14	1.357 ± 0.497
	AA	1	1.000 ± 0.000
g.28317295G>A	GG	74	1.419 ± 0.467
	GA	16	1.375 ± 0.500
g.28317368G>A	GG	12	1.500 ± 0.522
	AA	46	1.435 ± 0.501
	GA	32	1.344 ± 0.483
g.28317623C>T	CC	80	1.388 ± 0.490
	CT	10	1.600 ± 0.516
g.28317663A>C	AA	66	1.349 ± 0.480^b^
	AC	24	1.583 ± 0.504^a^
g.28317758G>A	GG	65	1.354 ± 0.482
	GA	22	1.500 ± 0.512
	AA	3	2.000 ± 0.000

**Note:**

Results are expressed as mean ± standard deviation. Different letters indicate significant differences (*p* < 0.05).

### Bioinformatics analysis of the *INHA* g.28317663A>C

**Figure 3:**
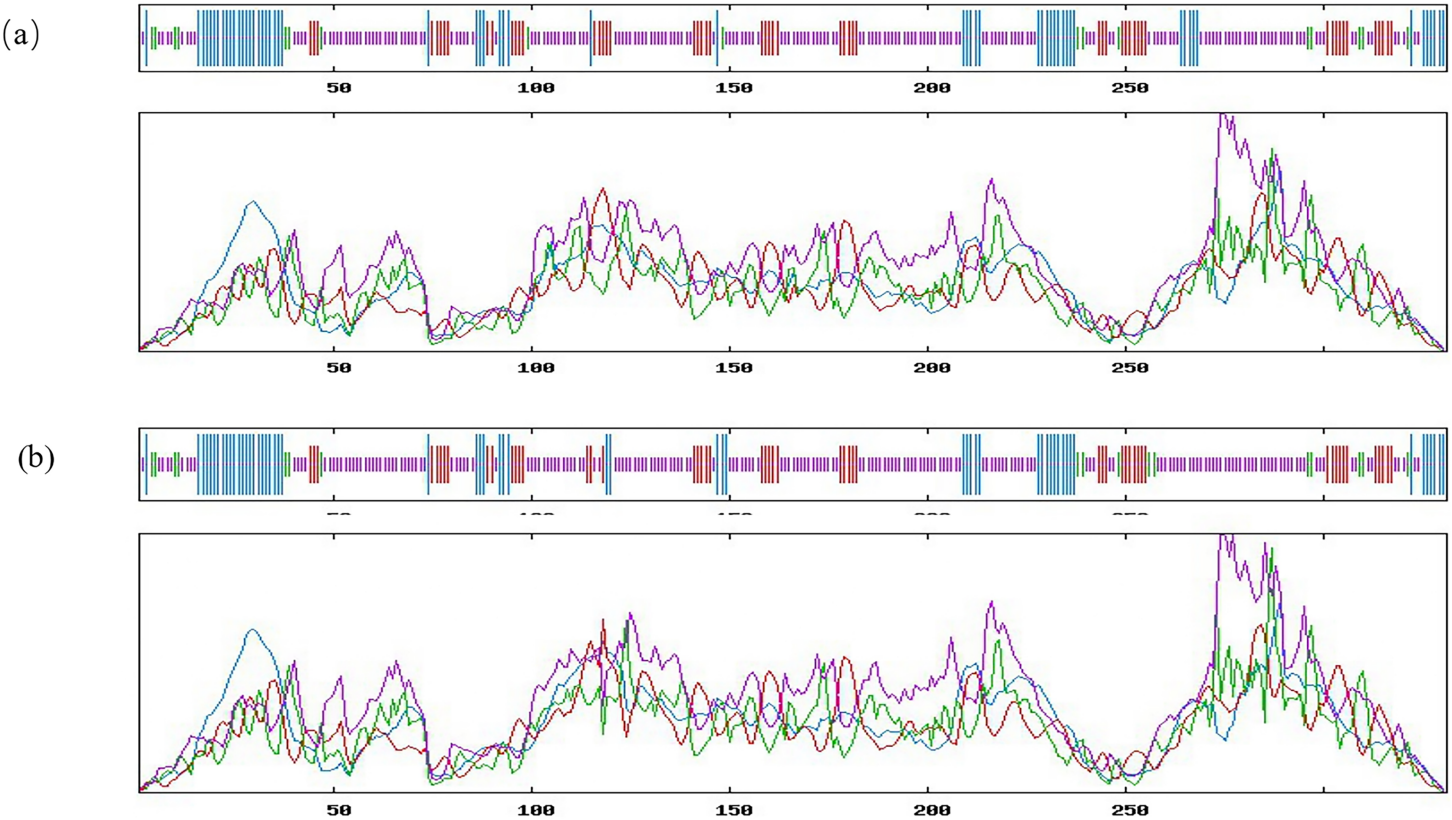
Bioinformatics analysis of the *INHA* gene with g.28317663A>C. Prediction of the secondary structure of the *INHA* protein of alleles A (A) and allele C (B). Blue, alpha helix; purple, extended strand; green, beta turn; orange, random coil.

**Figure 4:**
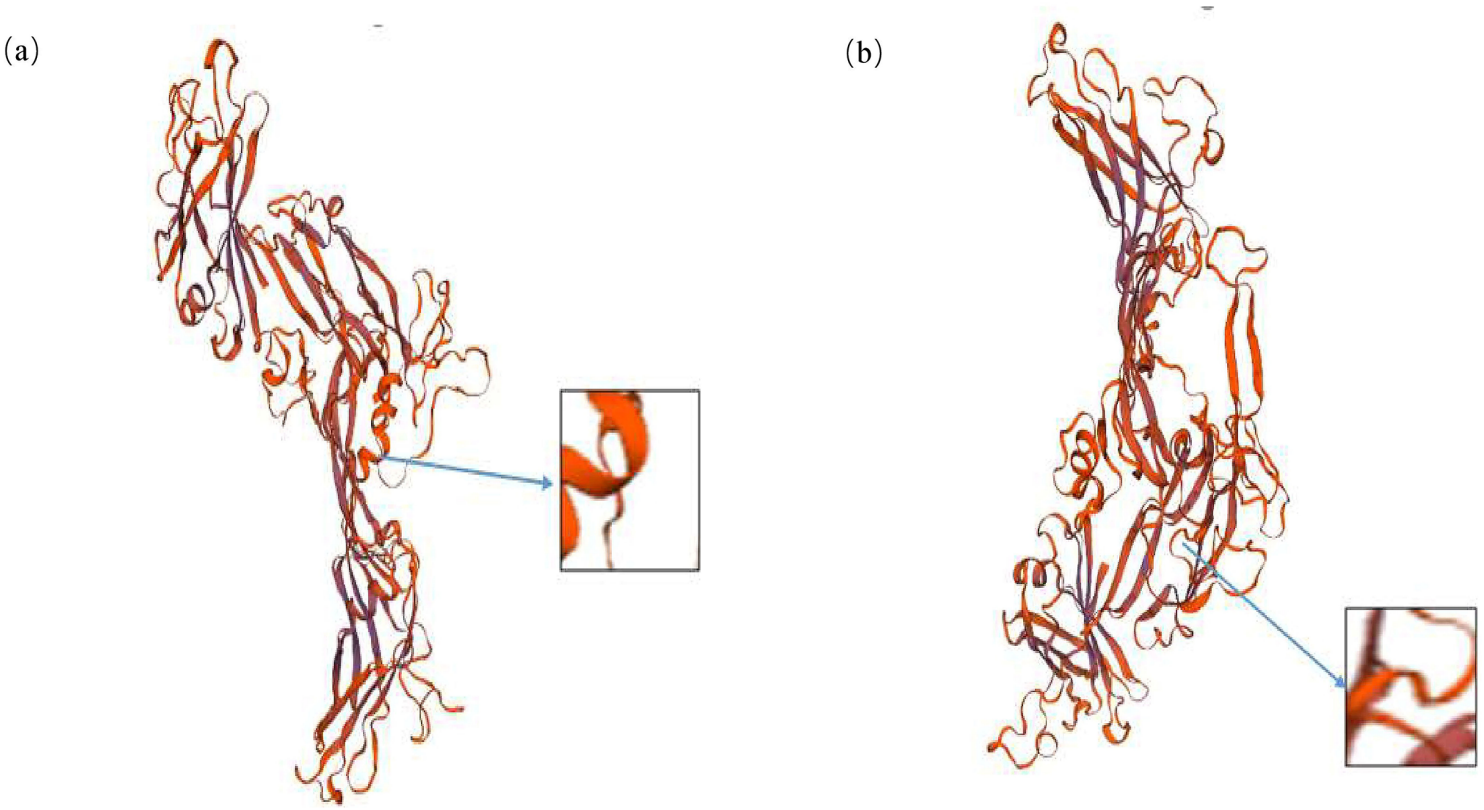
Bioinformatics analysis of the *INHA* gene with g.28317663A>C. Prediction of the tertiary structure of the *INHA* protein for alleles A (A) and allele C (B).

It is noteworthy that the mutation at g.28317663 loci leads to the change of amino acid sequence at the 316 site of inhibin A protein, which is Thr change Pro. PRABI was used to predict the protein secondary structure of alleles A and C at locus 316. The secondary structure of the alpha helix of allele A inhibin protein was 18.13%, that of the extended strand was 16.62%, that of the beta turn was 5.44%, and that of the random coil was 59.82%. The secondary structure of the alpha helix of allele C inhibin protein was 17.52%, that of the extended strand was 16.01%, that of the beta turn was 5.44%, and that of the Random coil was 61.03%. It was found that the missense mutation site changed the alpha helix, random coil, and extended strand in the secondary structure of the protein (Fig. 3). Using SWISS to predict the changes in the tertiary structure of the proteins of alleles A and allele C at locus 316, significant changes with g.28317663A>C mutation were observed (Fig. 4).

### Linkage disequilibrium and haplotype frequency

**Figure 5:**
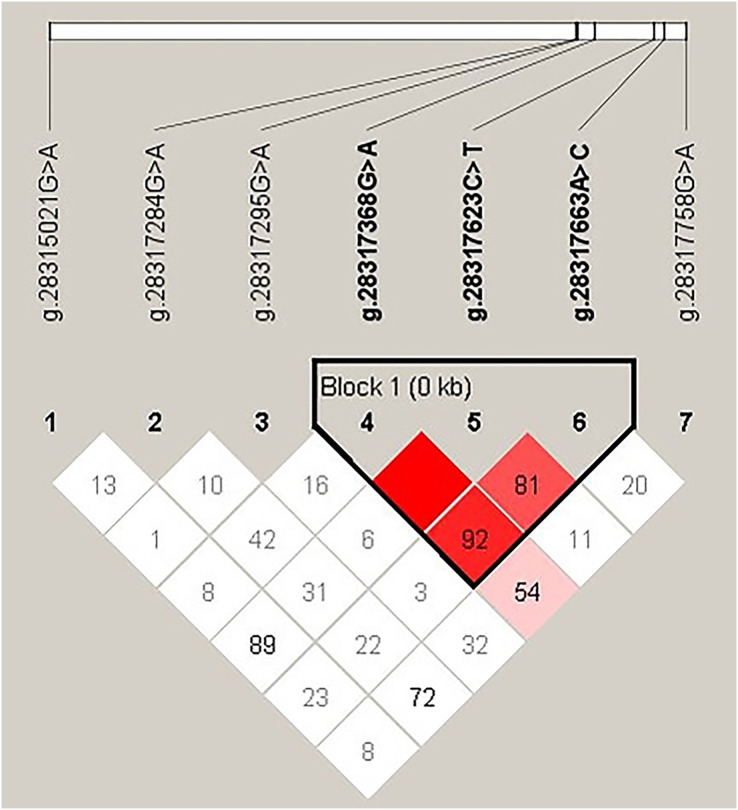
Disequilibrium of linkage among seven SNPS of *INHA* gene in Hainan black goats. The color of the square indicates the degree of linkage, the darker the color, the higher the degree of linkage; The value represents the strength of the correlation between sites (percentage).

In order to explore the linkage relationship among the seven SNPs in the *INHA* gene of Hainan black goats (Fig. 5), linkage disequilibrium parameters (D′ and r2) were used. The results of the linkage disequilibrium are showed in Table 5. Linkage disequilibrium was found between g.28317368G>A, g.28317623C>T, and g.28317663A>C of the *INHA* gene. In these three SNPs, the D′ values ranged from 0.817 to 1.000 and the r2 values from 0.079 to 0.264. When subject to a 1% haplotype frequency threshold (Haploview4.2), a total of four haplotypes were identified within three SNPs of the Hainan black goat *INHA* gene. With regard to haplotype frequency, the ACA haplotype was found to be the most common, observed at a frequency of 0.595, while the GTC haplotype displayed the lowest frequency, occurring at a frequency of 0.042 (Table 6).

**Table 5:** Estimated linkage disequilibrium for the SNPs identified in the *INHA* gene.

	D′	r^2^
g.28317368G>A/g.28317623C>T	1.000	0.079
g.28317368G>A/g.28317663A>C	0.921	0.264
g.28317623C>T/g.28317663A>C	0.817	0.220

**Table 6:** Estimates of haplotypes frequencies observed in the *INHA* gene.

Haplotype	g.28317368G>A	g.28317623C>T	g.28317663A>C	Haplotype frequency
Hap1 (ACA)	A	C	A	0.595
Hap2 (GCA)	G	C	A	0.260
Hap3 (GCC)	G	C	C	0.089
Hap4 (GTC)	G	T	C	0.042

### Association analysis of *INHA* gene haplotype and litter size in Hainan black goats

We analyzed the association between *INHA* gene haplotypes and litter size in Hainan black goats (Table 7). The results showed that there was no significant association between haplotypes and litter size.

**Table 7:** Association analysis of *INHA* gene haplotypes and litter size in Hainan black goats.

Haplotype	Number of samples	Litter size (Mean ± SD)
Hap1 (ACA)	77	1.407 ± 0.493
Hap2 (GCA)	58	1.448 ± 0.501
Hap3 (GCC)	23	1.608 ± 0.499
Hap4 (GTC)	7	1.714 ± 0.487
*p* value		0.135

## Discussion

Inhibin is an important inhibitor of pituitary gonadotropin secretion of *FSH* (Cai et al., 2015). Inhibin can competitively bind activing (*ACT*) receptor with *ACT* to weaken the action of *ACT*, thereby reducing the effect of *ACT* on the positive feedback regulation of pituitary *FSH* synthesis and secretion, and achieving the effect of negative regulation of *FSH* secretion (Gaccioli et al., 2018). *FSH* can stimulate the growth and development of follicles (Casarini & Crepieux, 2019). Decreased inhibin induces multiple ovulation and increase pituitary secretion of *FSH*, which appears to be the primary mechanism for stimulating additional follicle growth (Wang et al., 2012). Hence, the polymorphism of *INHA* gene involving the functional center of inhibin has the potential to affect the reproductive performance of animals. Previous studies have found that a large number of *INHA* gene mutations are closely related to reproductive system problems such as male infertility (Li et al., 2015), premature ovarian failure (Yoon et al., 2012), superovulation (Luo et al., 2019), follicular cysts (Li et al., 2016), and sperm quality (Rafaqat et al., 2020). Considering the above research results, we believe that the *INHA* gene is an important candidate gene for improving kidding in does. However, it is still unknown whether *INHA* gene polymorphism has an effect on the reproductive traits of Hainan black goats. Therefore, the relationship between SNPs of the *INHA* gene and the kidding of Hainan black goats was analyzed, helping to find useful molecular markers for breeding selection Hainan black goats.

Polymorphisms of the *INHA* gene have been verified in different animals. In this study, we found seven SNPs in this gene, including three new SNPs, which were g.28315021G>A, g.28317368G>A, and g.28317758G>A. Wu et al. (2009) identified 12 SNPs of the *INHA* gene in Boer goats, four of which were consistent with our study. In addition, studies in other animals have also found that this gene has higher polymorphisms, such as 12 SNPs being detected in the *INHA* gene of chickens (Cui et al., 2019) and three SNPs in the *INHA* gene in Murrah bulls (Chandra et al., 2020). Combined with our results, the *INHA* gene has abundant polymorphisms.

Polymorphisms of the *INHA* gene are related to reproductive traits in different animals. Studies have found that polymorphisms in the *INHA* gene are closely related to the reproductive traits of Holstein cows (Sang et al., 2011; Tang et al., 2011). *INHA* gene polymorphisms have been shown to be significantly correlated with litter size in several goat breeds. For example, Jining Grey goat does with the genotype *GA* having 0.79 (*p* < 0.01) more kids than those with the genotype *GG* in the g.28315680 locus of the *INHA* gene (Liu et al., 2017b). For g.28318073C>T of the *INHA* gene, Nigerian goat does with the genotype *CT* had 0.33 (*p* < 0.05) more kids than those with the genotype *CC* (Isa et al., 2017). These results are similar to those in the present study. In our study, for g.28317663A>C of the *INHA* gene, the litter size of the *AC* genotype was significantly higher than that of the *AA* genotype.

In this study, g.28317663A>C, which is a missense mutation, was significantly associated with litter size in Hainan black goats. *INHA* missense mutations have also been found to be significantly associated with litter size in Malabari goats (Pillai & Venkatachalapathy, 2020). Missense mutations cause amino acid changes that can alter protein properties (*e.g*., binding, expression, and protein stability) (Norn, André & Theobald, 2021). For example, studies have found that mutations in the *ASMT* and *ADAMTS1* genes can cause changes in protein structure and affect protein function, which may affect the litter size of goats (Hu et al., 2020). We speculate that the change of amino acids from Thr to Pro may affect the function of *INHA* protein by impacting the structure of secondary and tertiary proteins. This change may affect the secretion of pituitary *FSH*, and then affect the concentration of *FSH*, which may ultimately increase the litter size of Hainan black goats. However, the effect still needs further verification.

In this study, three SNPs of the *INHA* gene were found to be in linkage disequilibrium in Hainan black goats (Table 4) which include g.28317368G>A, g.28317623C>T, and g.28317663A>C of *INHA* genes. To the best of our knowledge, our study is the first to analyze the linkage disequilibrium of the SNPs of the *INHA* gene in Hainan black goats. In addition, linkage disequilibrium in the SNPs of this gene has also been found in Luhua chickens (Cui et al., 2019). If these polymorphisms are in linkage disequilibrium with genes that influence variation in reproductive traits, segregation based on marker alleles will result in phenotypic differences. Therefore, the linkage disequilibrium phenomenon needs to be considered when this gene polymorphism is applied to the breeding of goats with a high litter size.

Due to the ancestral structure captured in the distribution of haplotypes, the haplotypes show more influence than the single SNPs for important trait associations (Akey, Jin & Xiong, 2001). Although no significant correlation was found between haplotype and litter size in the haplotype analysis, we found that H3 and H4 enriched with the g.28317663 C allele had a higher litter size than H1 and H2. The g.28317663 C allele may be related to litter size, which supports g.28317663A>C being a useful genetic marker affecting the litter size of Hainan black goats.

## Conclusions

In this study, a total of seven SNPs loci were found in the *INHA* gene of Hainan black goats, among which g.28317663A>C was significantly associated with litter size. Mutation at this site will cause changes in amino acids from Thr to Pro, affecting the secondary and tertiary structure of *INHA* protein. The *INHA* gene is one of the genes that affect the reproductive capacity of Hainan black goats, and the g.28317663A>C loci may be a potential genetic marker for future breeding. It will be nevertheless necessary to expand the sample size to further study the impact of *INHA* gene polymorphism on the litter size of Hainan black goats.

## Supplemental Information

10.7717/peerj.15381/supp-1 Supplemental Information 1 The electrophoresis result of PCR amplified by primer *INHA*-5. The primer *INHA*-5 was used to amplify 6 SNPs in exon 2 of the *INHA* gene

10.7717/peerj.15381/supp-2 Supplemental Information 2 Raw data of Tables 3, 4, 7.

10.7717/peerj.15381/supp-3 Supplemental Information 3 Full (21-point) ARRIVE 2.0 checklist.

## Additional Information and Declarations

### DNA Deposition

The following information was supplied regarding the deposition of DNA sequences: The data is available at NCBI: OQ461736-OQ462157.

### Data Availability

The following information was supplied regarding data availability: The raw sequencing data is available at Figshare: Li, Lianbin (2023): GOATS.rar. figshare. Dataset. https://doi.org/10.6084/m9.figshare.22140080.v1.
