# Supplementary material for: Association between INHA gene polymorphisms and litter size in Hainan black goats
Source: PeerJ. 2023 May 9;11:e15381. doi: 10.7717/peerj.15381 (PMC10178212; doi:10.7717/peerj.15381)
Supplement: Supplemental Information 1 — The primer INHA-5 was used to amplify 6 SNPs in exon 2 of the INHA gene [file peerj-11-15381-s001.pdf]

Supplementary Material

Supplementary Figure S1

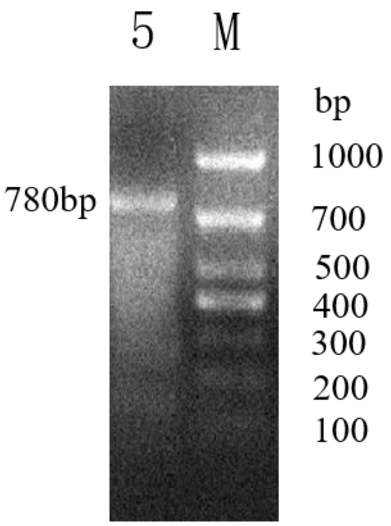

Supplementary Figure S1. The electrophoresis result of PCR amplified by primer *INHA*-5. The primer *INHA*-5 was used to amplify 6 SNPs in exon 2 of the *INHA* gene.
