# Supplementary material for: Association between INHA gene polymorphisms and litter size in Hainan black goats
Source: PeerJ. 2023 May 9;11:e15381. doi: 10.7717/peerj.15381 (PMC10178212; doi:10.7717/peerj.15381)
Supplement: Supplemental Information 3 [file peerj-11-15381-s003.pdf]

The ARRIVE Essential 10<br>Association between INHA Gene Polymorphisms and litter size in Hainan black goats		
Item	Recommendation	Section/line number, or reason for not reporting
Study design	1 Jugular blood samples of 211 Hainan black does were collected from Hainan Chuxin Animal Husbandry Co,. Ltd. (Ding'an, Hainan, China) in two different locations. The goat is normally raised in the goat house by the company, and the company is responsible for its breeding and feeding management. This study only requires blood collection. There were 90 Hainan black does with clear lambing records for the full year of 2019, of which 37 had two lambs per litter and 53 had single lambs per litter. The remaining 121 Hainan black does were randomly selected in the farms.	62-69
Sample size	A total of 211 healthy Hainan black goats with similar body conditions were selected, among which 90 Hainan black goats had a clear record of a lambing. The remaining 121 Hainan black does were randomly selected in the farms.	62-64<br>66-69
Inclusion and exclusion criteria	The inclusion and exclusion criteria were ewes' health and reproductive performance. Healthy ewes that have already lambed are selected, and vice versa.	
Randomisation	There were 90 Hainan black does with a clear record of a lambing, of which 37 had two lambs per litter and 53 had single lambs per litter. The remaining 121 Hainan black does were randomly selected in the farms.	66-69
Blinding	We didn't do a group design for goats.	
Outcome measures	1. PCR products were electrophoretic on 2% agarose gel. Observing the target band under the gel imager.<br>2. The gene sequence was detected by Shanghai Shengong Bioengineering Co., LTD., and then visualized using SnapGene software. The genotype was deter-mined by sequencing the peak map.	
Statistical methods	Popgen32 software was used to count the genotype frequency, allele frequency, observed heterozygosity (Ho), expected heterozygosity (He), effective allele number (Ne), and polymorphism information content (PIC), and to conduct a Hardy	88-98

	Weinberg equilibrium test. Haploview4.2 software was used to analyze the haplotypes and link-age disequilibrium (LD) of the SNPs. Haplotype identification was achieved with the Four Gamete Rule. At the same time, the general linear model (GLM) program of SPSS 19.0 software was used to analyze the influence of genotypes and haplotypes on the litter size of Hainan black goats. The model was as follows: $$Yijn = \mu + Gi + eijn$$ where Yijn is the litter size phenotype of the individual Hainan black goats, μ is the population mean, Gi is the effect of genotype, and eijn is the random error effect.	
Experimental animals	Hainan black goat breeds were selected in this experiment, all of them were female, and their age and weight were unknown. All ewes are from Hainan Chuxin Animal Husbandry Co Ltd. (Ding'an, Hainan, China), healthy, without genetic modification and previous experiments.	
Experimental procedures	211 Hainan black goats were collected blood from jugular vein, then genomic DNA was extracted, and finally INHA gene was amplified.	
Results	It was found that there were 7 SNPs in INHA gene in Hainan Black Goats.	

The Recommended Set<br>Association between INHA Gene Polymorphisms and litter size in Hainan black goats		
Item	Recommendation	Section/line number, or reason for not reporting
Abstract	In order to extract genomic DNA from 211 Hainan black goats, blood was collected by jugular vein sampling, and genomic DNA of Hainan black goats was extracted by blood genomic DNA extraction kit.	
Background	With the continuous development of goat breeding industry, blood collection has become one of the important means to monitor the quality and health of goats, and blood collection from jugular vein is one of the routine blood collection methods of goats.	
Objectives	Genomic DNA is extracted by collecting blood from the jugular vein.	
Ethical statement	All conducted procedures were approved by the Ethics Committee	

	at Hainan University (No. HNUAUCC-2022-000121).	
Housing and husbandry	We did not conduct experiments involving animal husbandry. This study also did not buy goats, we just went to goat farms to collect blood. Only goat genomes were obtained from these blood samples. Jugular vein blood sampling in goats also does not require anesthesia. There is no harm to the goat after blood collection. The goats after blood collection will be raised normally by the breeding company.	
Animal care and monitoring	We did not conduct experiments involving animal husbandry. This study also did not buy goats, we just went to goat farms to collect blood. Only goat genomes were obtained from these blood samples. Jugular vein blood sampling in goats also does not require anesthesia. There is no harm to the goat after blood collection. The goats after blood collection will be raised normally by the breeding company.	
Interpretation/ scientific implications	This study is only for jugular vein blood collection.	
Generalisability/ translation	This study is only for jugular vein blood collection and does not involve this part.	
Protocol registration	This study is only for jugular vein blood collection and does not involve this part.	
Data access	In this study, the sequencing data of DNA fragments generated by the first-generation sequencing technology (Sanger sequencing) were uploaded to figshare as raw data. The link is https://figshare.com/search?q=10.6084%2Fm9.figshare.21617412	
Declaration of interests	All authors declare no conflicts of interest. This research was funded by Academician Innovation Platform Project of Hainan Province (No. YSPTZX202153), Innovation Platform for Academician of Hainan Province, China Mordern Agriculture Research System MOF and MARA (CARS-38) and the Natural Science Foundation of Hainan Province (No.2019RC090).	
